# Interferon-γ treatment in vitro elicits some of the changes in cathepsin S and antigen presentation characteristic of lacrimal glands and corneas from the NOD mouse model of Sjögren’s Syndrome

**DOI:** 10.1371/journal.pone.0184781

**Published:** 2017-09-13

**Authors:** Zhen Meng, Wannita Klinngam, Maria C. Edman, Sarah F. Hamm-Alvarez

**Affiliations:** 1 Department of Pharmacology and Pharmaceutical Sciences, School of Pharmacy, University of Southern California, Los Angeles, California, United States of America; 2 Department of Ophthalmology, USC Roski Eye Institute and Keck School of Medicine, University of Southern California, Los Angeles, California, United States of America; Mie University Graduate School of Medicine, JAPAN

## Abstract

Inflammation and impaired secretion by lacrimal and salivary glands are hallmarks of the autoimmune disease, Sjögren’s Syndrome. These changes in the lacrimal gland promote dryness and inflammation of the ocular surface, causing pain, irritation and corneal damage. The changes that initiate and sustain autoimmune inflammation in the lacrimal gland are not well-established. Here we demonstrate that interferon-γ (IFN-γ) is significantly elevated in lacrimal gland and tears of the male NOD mouse, a model of autoimmune dacryoadenitis which exhibits many ocular characteristics of Sjögren’s Syndrome, by 12 weeks of age early in lacrimal gland inflammation. Working either with primary cultured lacrimal gland acinar cells from BALB/c mice and/or rabbits, in vitro IFN-γ treatment for 48 hr decreased expression of Rab3D concurrent with increased expression of cathepsin S. Although total cellular cathepsin S activity was not commensurately increased, IFN-γ treated lacrimal gland acinar cells showed a significant increase in carbachol-stimulated secretion of cathepsin S similar to the lacrimal gland in disease. In vitro IFN-γ treatment did not increase the expression of most components of major histocompatibility complex (MHC) class II-mediated antigen presentation although antigen presentation was slightly but significantly stimulated in primary cultured lacrimal gland acinar cells. However, exposure of cultured human corneal epithelial cells to IFN-γ more robustly increased expression and activity of cathepsin S in parallel with increased expression and function of MHC class II-mediated antigen presentation. We propose that early elevations in IFN-γ contribute to specific features of ocular disease pathology in Sjögren’s Syndrome.

## Introduction

Sjögren’s Syndrome (SS) is a chronic autoimmune disease that primarily affects lacrimal glands (LG) and salivary glands (SG), leading to reduced production and altered composition of tears and saliva and, respectively, ocular surface inflammation/corneal damage and compromised oral health [1]. Initial events in SS are characterized by inflammation and loss of secretory function in LG and SG. While the molecular and cellular mechanisms responsible for initiation and perpetuation of these two changes are largely unclear, early changes in acinar cell responses to altered cytokine levels may play a role.

Th1 and Th17 cytokines are implicated in the pathogenesis of SS; although results are variable, Th1 cytokines (e.g. interferon-γ (IFN-γ), interleukin (IL)-2) and Th17 cytokines (e.g. tumor necrosis factor (TNF)-α and IL-17) are highly expressed in SS patients [2, 3]. Specifically, pro-inflammatory cytokines such as IL-1α, IL-2, IFN-γ, IL-6, and TNF-α are increased in SG of SS patients [4], in parallel with increased major histocompatibility complex (MHC) class II molecules in SG acinar cells [5, 6]. Many studies have shown that MHC class II expression is upregulated in SG epithelial cells from SS patients compared to healthy controls, while a role for IFN-γ in increased expression of antigen-presenting molecules [6, 7] and ICAM1 [8] on SG acinar cell plasma membranes and induction of antigen presentation is supported [9]. Classical actions of IFN-γ include not only the induction of MHC class II-mediated CD4+ T cell activation by presentation of exogenous antigenic peptides but also induction and activation of this pathway in non-professional antigen-presenting cells [10, 11]. Fewer studies have been conducted in LG acinar cells (LGAC), although one previous study found that MHC class II expression was elevated in epithelial cells of cadaver lacrimal glands exhibiting a higher T cell infiltration [12]. In vitro studies have implicated increased MHC class II expression in the process of lymphocytic proliferation driven by LGAC [12].

Cytokines such as IFN-γ may further contribute to exocrine gland pathogenesis by interacting directly with glandular epithelial cells to alter additional functions beyond induction of antigen presentation. The principal function of acinar cells within exocrine tissue is secretion of proteins and fluid. Although lymphocytic infiltration in the exocrine glands is considered a hallmark of SS, many patients suffered from secretory dysfunction without corresponding massive lymphocytic infiltration and tissue destruction [13, 14]. Pro-inflammatory cytokines, for example, IFN-γ, are highly expressed in glands of individuals with sicca symptoms who lack lymphocytic infiltration [15]. It is possible that pro-inflammatory cytokines have a direct effect on the secretory function of acinar cells independent of autoimmune inflammation.

Altered protein trafficking and secretion dysfunction of LG has been reported in SS patients and in animal models of SS [16–18]. Our previous work evaluating autoimmune LG inflammation (dacryoadenitis) in a murine model of SS, the non-obese diabetic (NOD) mouse, revealed that increased expression and activity of a lysosomal protease, cathepsin S (CTSS) in the LG paralleled the onset of lymphocytic infiltration [19, 20]; moreover, CTSS was increased not only in LGAC but was secreted in increased amounts into tears of the NOD mice. Our clinical study has confirmed that CTSS activity is significantly enhanced in SS patient tears relative to tears of patients with other dry eye or non-SS autoimmune diseases [18]. Increased tear CTSS may not only denote LG inflammation but promote ocular surface inflammation through diverse mechanisms including extracellular matrix degradation and induction of multiple inflammatory pathways [21, 22]. Our recent study has linked changes in the expression and activity of secretory Rab proteins, small GTPases serving as sorting codes for vesicular trafficking, to the increased tear CTSS that occurs in autoimmune dacryoadenitis [17]. Specifically, loss of Rab3D was associated with secretion of a more proteolytic tear film containing increased CTSS. Loss of Rab3D is also a feature of LGAC and SG acinar cells from SS patients [16, 23].

Here, we identified IFN-γ as a major pro-inflammatory cytokine elevated in LG and tears of the male NOD mouse model of SS early in autoimmune dacryoadenitis. We hypothesized that several possible changes in LGAC may occur in early autoimmune dacryoadenitis in response to elevated IFN-γ in the LG, each related to triggering and perpetuation of autoimmune disease. These changes include 1) Reduced expression/function of Rab3D; 2) Increased secretion of CTSS to tears; 3) Increased expression of CTSS, MHC class II and other effectors of antigen presentation; and 4) Increased functional processing of antigens. We tested the effects of IFN-γ on each of these indicators of early acinar cell dysfunction and immunopathology in vitro in cultured LGAC, and also explored the consequent effects in vitro of exposure of corneal epithelial cells to IFN-γ. Our results show that IFN-γ treatment reduces expression of Rab3D and increases stimulated secretion of CTSS in LGAC, but does not notably increase expression of components of MHC class II-mediated antigen presentation nor dramatically increase this function in LGAC. However, IFN-γ exposure does elicit features of immunopathology in cultured corneal epithelial cells, specifically increasing expression of components of MHC class II-mediated antigen presentation and functional antigen processing.

## Results

### Pro-inflammatory cytokines are increased in LG of male NOD mice, a model of autoimmune dacryoadenitis exhibiting characteristic features of SS-associated dry eye

The NOD mouse spontaneously develops autoimmune inflammation and loss of secretory function of the LG (dacryoadenitis) and SG (sialoadenitis), as well as systemic features of SS such as increased serum autoantibodies [24, 25]. The pattern of disease development is markedly different between males and females. Dacryoadenitis but not sialoadenitis is present in male NOD mice by 12 weeks of age [26]. As well, by 12 weeks of age, male NOD mice exhibit changes in composition of tears such as increased CTSS activity that are characteristic of SS patients [17, 18]. In contrast, female NOD mice develop sialoadenitis by 12 weeks of age [27] but barely exhibit dacryoadenitis even by 20 weeks of age [28]. Since our focus is on the origin of the changes in LGAC that perpetuate the autoimmune LG and ocular surface inflammation underlying SS-associated dry eye, our in vivo mouse work utilizes male NOD mice and healthy control BALB/c mice at 12 weeks. The levels of ten different cytokines, many linked with SS in models of disease or in patients [2, 4], were assayed in lysates from male NOD and BALB/c mouse LG. All cytokines evaluated were elevated in NOD mouse LG compared with the BALB/c control mouse LG (Table 1). Among the cytokines analyzed, IFN-γ, IL-10, IL-1β, IL-2, KC/GRO, and TNF-α levels were increased to the greatest extent. We selected IFN-γ as our major focus since it increases expression of CTSS in different cells including macrophages, alveolar epithelial cells, hepatic stellate cells [29–31] and is a known inducer of CTSS expression associated with increases in other components of MHC class II-mediated antigen presentation, each of which has been linked to autoimmune dacryoadenitis [9, 32].

**Table 1:** Inflammatory cytokine levels are significantly increased in male NOD mouse LG as detected by MSD^®^ multiplex assay.

	**IFN-γ**	**IL-10**	**IL-12 p70**	**IL-1β**	**IL-2**
**BALB/c**	**5.859 ± 1.356**	**238.1 ± 38.78**	1914 ± 711.3	**43.51 ± 7.685**	**24.75 ± 1.711**
**NOD**	**263.8 ± 82.61**	**521.7 ± 81.85**	2389 ± 232.9	**272.5 ± 92.63**	**80.19 ± 19.89**
***P* value**	0.0286*	0.0286*	0.3429	0.0286*	0.0286*
	**IL-4**	**IL-5**	**IL-6**	**KC/GRO**	**TNF-α**
**BALB/c**	32.33 ± 9.635	24.40 ± 2.578	1113 ± 256.9	**3900 ± 942.2**	**194.7 ± 66.61**
**NOD**	39.37 ± 7.626	35.02 ± 2.573	1373 ± 241.5	**14990 ± 2411**	**781.5 ± 194.1**
***P* value**	0.8857	0.0571	0.2000	0.0268*	0.0268*

Ten inflammatory cytokines were measured in NOD and BALB/c mouse LG lysates. Cytokine concentrations are shown as mean ± standard error of the mean (SE) in 10^−5^ pg per microgram total protein. N = 4.

*, *P* ≤ 0.05. Bold, significantly changed.

### IFN-γ exposure alters gene and protein expression of Rab3D and increases stimulated secretion of CTSS, similar to patterns seen in male NOD mice with autoimmune dacryoadenitis

**Fig 1:**
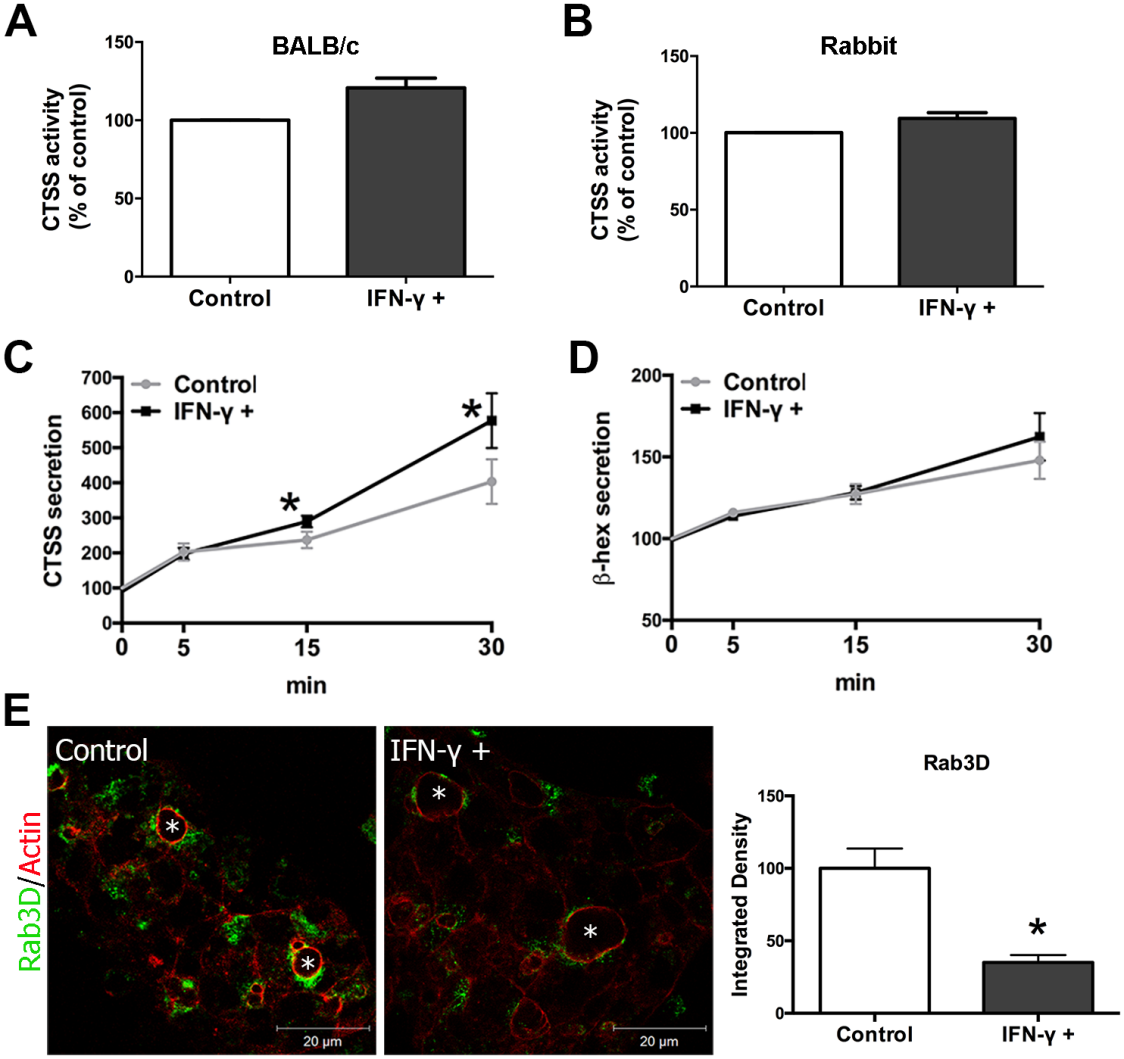
Changes in CTSS secretion and Rab3D expression in rabbit LGAC exposed to IFN-γ. **(A)** Activity of CTSS in lysates from untreated and mouse IFN-γ-treated (200 U/ml, 48 hr) BALB/c mouse LGAC lysates was measured with a commercial kit. CTSS activity was measured as relative fluorescence units (RFU) per μg total protein. Data are presented as relative values to those from non-treated cells, which were arbitrarily set as 100. N = 3. **(B)** Activity of CTSS in lysates from untreated and rabbit IFN-γ-treated (200 ng/ml, 48 hr) rabbit LGAC lysates. N = 3. **(C)** Activity of CTSS secreted into culture medium was measured from untreated and rabbit IFN-γ-treated rabbit LGAC after CCh stimulation for 5, 15 or 30 min. After CCh stimulation in untreated LGAC (control), CTSS secretion was increased significantly. However, after 15 min or 30 min CCh stimulation, CTSS secretion from IFN-γ-treated LGAC was significantly higher than that detected from untreated LGAC (*) (*P* = 0.0265 and 0.0009, respectively). CTSS activity was measured as relative fluorescence units per unit volume of medium. IFN-γ was added at 200 ng/ml for 48 hr. Data are presented as relative values to those from basal secretion of non-treated cells, which were arbitrarily set as 100. N = 6. **(D)** β-hexosaminidase (β-hex) secretion was increased significantly after CCh stimulation as we have reported, but no differences were detected between the IFN-γ-treated and untreated groups. N = 6. **(E)** Rab3D expression detected by immunofluorescence was modestly diminished in rabbit LGAC treated with IFN-γ at 200 ng/ml for 48 hr. *P* = 0.0018. *, lumen. Representative images are shown from three experiments. Quantification was done as in Methods with 9–15 ROIs from each experiment.

In previous studies exploring changes in tear protein secretion in male NOD mice with autoimmune dacryoadenitis, decreased levels of the secretory protein effector, Rab3D, were observed concomitant with increased CTSS gene expression and CTSS activity in tears in response to LG stimulation [17, 19]. In order to investigate whether IFN-γ contributed to these changes in LGAC related to secretory dysfunction, we treated cultured primary LGAC isolated from male BALB/c mice, the control for the NOD strain, with different doses (100, 200, 400 U/ml) and durations of treatment (1, 2, 4, 8, 24, 48 hr) of IFN-γ. We identified a dose of 200 U/ml of IFN-γ as the one associated with maximal changes in CTSS (increased) and Rab3D (decreased) gene expression at 48 hr, reflective of the diseased LG in situ. Since the BALB/c mouse is commonly used in comparison to NOD mice [19, 33], we continued detailed analysis in this model. In BALB/c mouse LGAC, IFN-γ treatment slightly but significantly decreased Rab3D gene expression while modestly but significantly increasing CTSS gene expression after 48 hr treatment (Table 2). Gene expression of other cathepsins that are present in lysosomes and implicated in antigen presentation in other cells [30, 34, 35] were also tested. Cathepsin L gene expression was significantly decreased by IFN-γ treatment while cathepsin B and D gene expression levels were not affected. The gene expression of the endogenous inhibitor of cathepsin S, cystatin C, was significantly downregulated by IFN-γ (Table 2). However, the total cellular CTSS activity did not show a significant increase with IFN-γ treatment (Fig 1A).

**Table 2:** Changes in gene expression of Rab3D, cathepsins and cystatin C in primary LG acinar cells from BALB/c mouse after IFN-γ treatment.

Gene	*Rab3d*	*Ctss*	*Ctsd*	*Ctsl*	*Ctsb*	*Cst3*
**RQ**	**0.83 ± 0.05**	**2.20 ± 0.28**	0.92 ± 0.06	**0.38 ± 0.05**	0.99 ± 0.03	**0.69 ± 0.06**
***P* value**	0.0152*	0.0022*	0.3874	0.0022*	0.3052	0.0022*

IFN-γ treatment was at 200 U/ml. *Cst3*, cystatin C. N = 3–8. RQ, relative quantity, is shown as mean ± SE.

*, *P* ≤ 0.05. Bold, significantly changed.

Since downregulation of Rab3D and upregulation of CTSS expression is associated with increased CTSS activity in stimulated tears of the male NOD mouse model of SS [17], to determine whether IFN-γ exposure could promote comparable changes in regulated CTSS secretion, we assayed its stimulated secretion in parallel with the abundant tear protein, β-hexosaminidase. We utilized primary rabbit LGAC for this in vitro study, since the larger size of the LG (400 mg versus 10 mg for mouse LG) resulted in a greater yield of acinar cells, enabling us to significantly reduce animal numbers from the twenty mice that would be required for a single mouse LGAC secretion experiment. Rabbit LGAC show comparable increases in gene expression of CTSS, with exposure to IFN-γ (S1 Table), but like mouse LGAC lysate, IFN-γ-treated rabbit LGAC lysates did not show a notable increase in CTSS activity (Fig 1B). However, CTSS secretion was significantly increased in response to the secretagogue, carbachol (CCh) in IFN-γ-treated cells after 15 min and 30 min of stimulation (Fig 1C); β-hexosaminidase secretion was unaffected by IFN-γ (Fig 1D), similar to results reported previously in male NOD mice and in LGAC in vitro in which Rab3D function is inhibited [17]. The magnitude of the increased CTSS release stimulated by CCh seen in vitro was not of the magnitude seen in vivo in the male NOD mouse (~ 5-fold) compared to BALB/c [17] or in SS patients (~ 40-fold) compared to healthy controls and (~ 4-fold) compared to patients with other autoimmune disease [18]. The enrichment of Rab3D immunofluorescence detected beneath apical/luminal regions was modestly decreased after IFN-γ treatment (Fig 1E), similar to the changes seen in the male NOD mouse LG, although the increased detection of Rab3D at basolateral membranes seen in NOD mouse LG was not noted [17].

### IFN-γ exposure promotes increased expression of some MHC class II-related molecules in vitro in LGAC comparable to patterns in NOD mice

**Fig 2:**
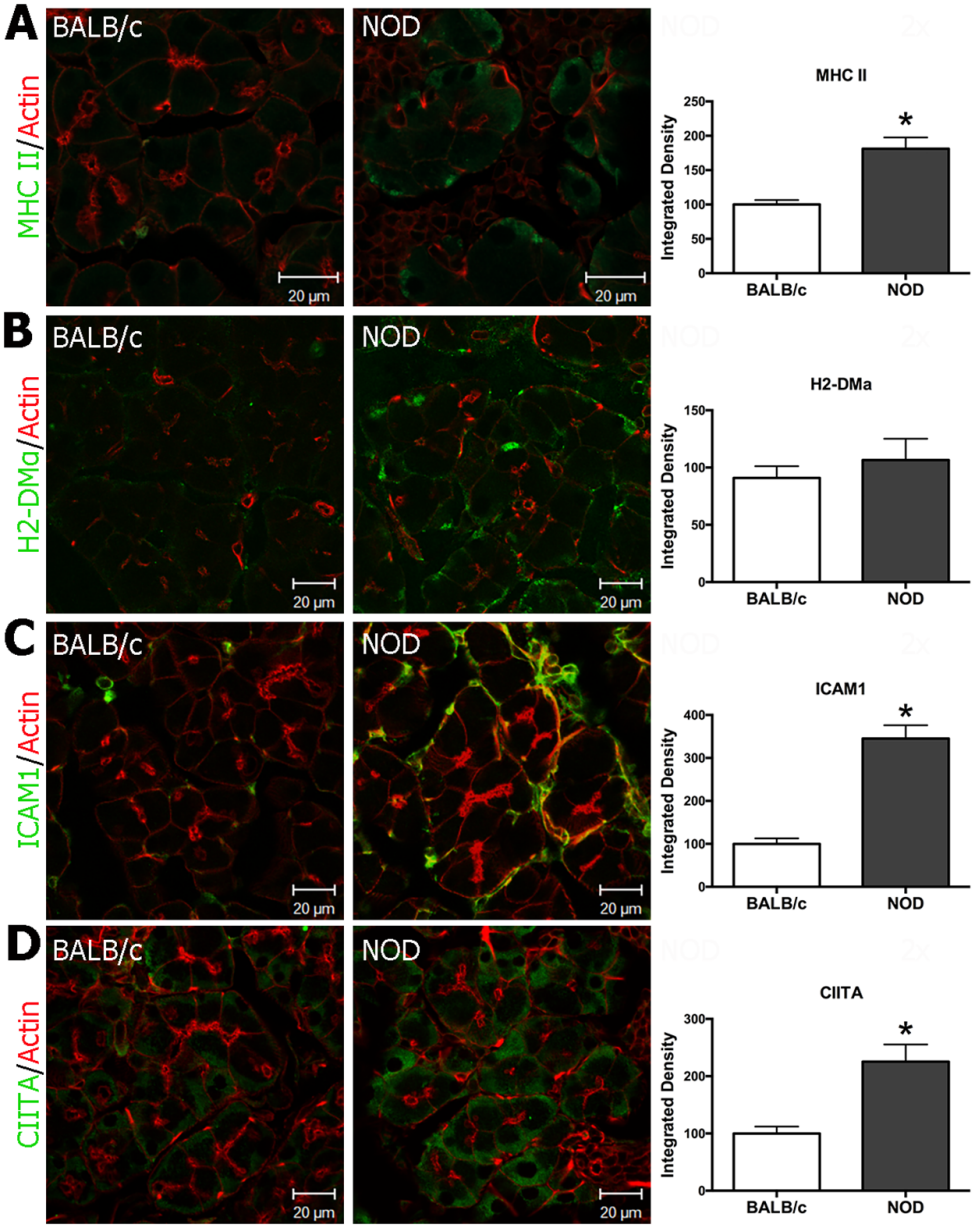
MHC class II, H2-DMa, ICAM1 and CIITA expression is increased in male NOD mouse LG acinar cells. Immunofluorescence was performed with LG from 12 weeks male BALB/c and NOD mice using anti-MHC II, anti-H2-DMa anti-ICAM1, and anti-CIITA antibodies plus appropriate fluorescently-labeled secondary antibodies as in Methods. Expression of MHC class II (MHC II) **(A)**, ICAM1 **(C)**, and CIITA **(D)** was significantly higher in NOD mouse LGAC with *P* <0.0001. However, H2-DMa **(B)** expression did not show a significant increase in NOD mouse LGAC. MHC II, H2-DMa and CIITA were present in a punctate distribution in the basolateral region of the acinar cells while ICAM1 was detected on the basolateral membrane of NOD mouse LGAC. Representative images are shown from three experiments. Quantification was done as in Methods with 7–15 ROIs from each experiment for each marker.

MHC class II molecules are present in LG and SG acinar cells from SS patients [5, 12]. SG acinar cells can be converted into antigen presenting cells by stimulation with IFN-γ [9]. We first tested gene expression levels of components of MHC class II-mediated antigen presentation in isolated acinar cells from LG from male NOD mice using laser capture microdissection (S1 Fig). The gene expression levels of CIITA (*Ciita*), MHC class II complex I-A dimer (*H2-Ab*), H2-DM molecule (*H2-DMa*), invariant chain (*Cd74*) and ICAM1 (*Icam1*) were all significantly increased in NOD mouse LGAC (Table 3). Protein expression levels of MHC class II, ICAM1 and CIITA were also increased as indicated by analysis of their signal by immunofluorescence in LG sections (Fig 2A, 2C and 2D). Interestingly, H2-DMa expression did not show a significant increase in NOD LGAC (Fig 2B). These results suggest that expression of molecules involved in MHC class II-mediated antigen presentation are increased in LGAC during development of autoimmune dacryoadenitis, suggestive of the conversion of LG acinar cells to antigen presenting cells as seen for acinar cells from SG.

**Table 3:** Changes in gene expression of MHC class II-related molecules in LG acinar cells of male NOD mouse compared with BALB/c.

Gene	*Ciita*	*H2-Ab1*	*H2-DMa*	*Cd74*	*Icam1*
**RQ**	**11.46 ± 3.71**	**7.05 ± 0.90**	**4.03 ± 1.01**	**162.90 ± 35.40**	**5.86 ± 1.18**
***P* value**	0.0485*	<0.0001*	0.0436*	0.0025*	0.0159*

Acinar cells were isolated from male NOD and BALB/c mouse LG by laser capture microdissection. N = 3–6. RQ, relative quantity, is shown as mean ± SE.

*, *P* ≤ 0.05. Bold, significantly changed.

**Fig 3:**
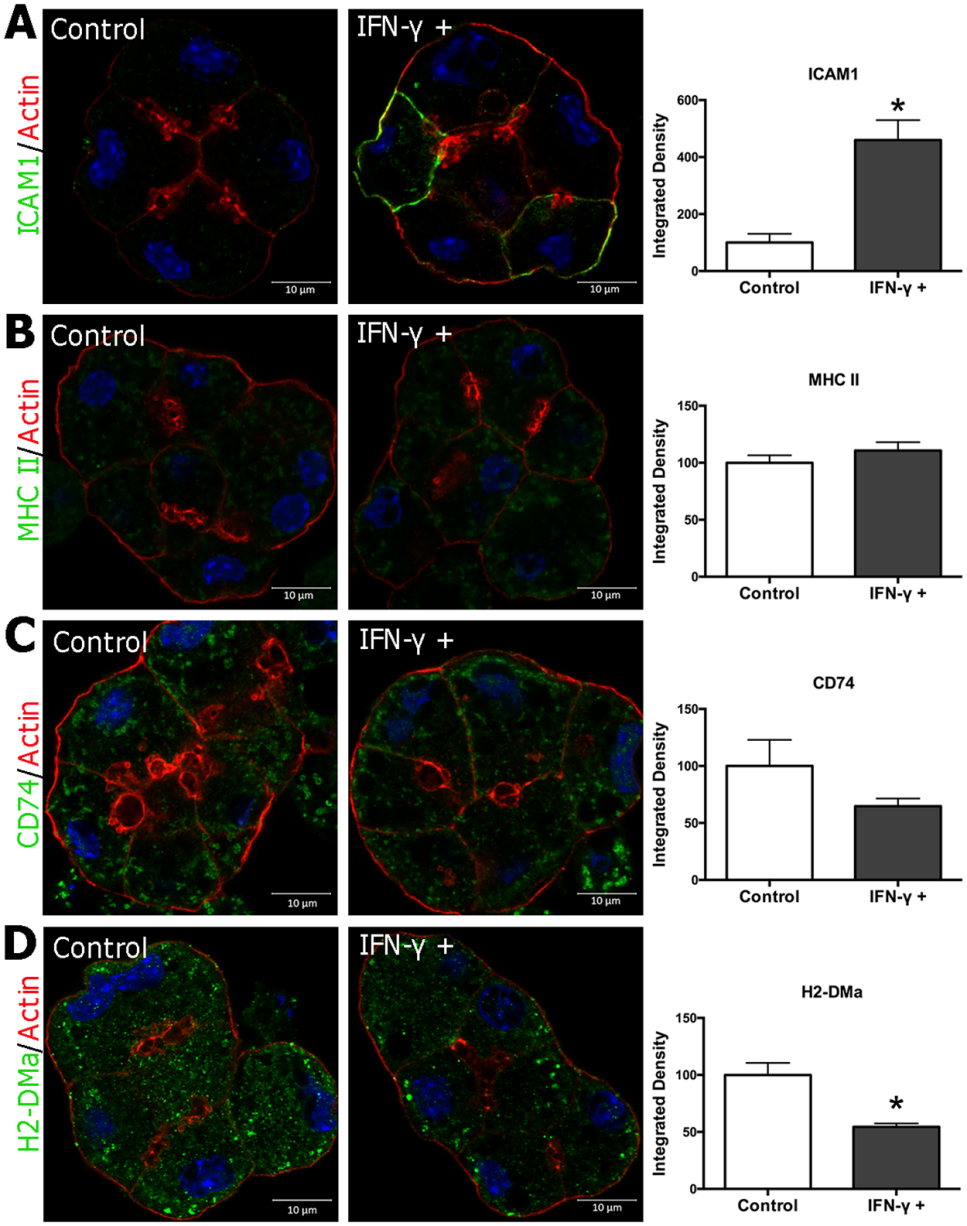
ICAM1 expression is increased as detected by immunofluorescence in mouse primary LG acinar cells treated with IFN-γ. Cultured BALB/c mouse LGAC were incubated for 48 hr with or without IFN-γ (200 U/ml). **(A)** ICAM1 was detected by indirect immunofluorescence. No profound changes in MHC class II (MHC II) **(B)**, invariant chain (CD74) **(C)**, or H2-DMa **(D)** expression were detected by immunostaining. Results were obtained from three experiments. Quantification was done as in Methods with 3–4 ROIs from each experiment for each marker.

To explore whether the increased levels of MHC class II molecules could be induced in vitro by IFN-γ, we assayed gene expression levels in IFN-γ-treated cultured LGAC from male BALB/c mice. Gene expression of the following MHC class II-related molecules, *H2-ab1*, *H2-dma*, *Cd74* and *Ciita*, and *Icam1* all were all strongly increased through 48 hr of exposure to IFN-γ (Table 4). Increased ICAM1 protein expression was also detected by indirect immunofluorescence and quantitative image analysis (Fig 3A). However, no profound increases in immunofluorescence signal associated with expression of other components of the MHC class II-mediated antigen presentation machinery were detected in the IFN-γ-treated mouse LGAC (Fig 3B–3D).

**Table 4:** Changes in gene expression of MHC class II-related molecules in primary LG acinar cells from BALB/c mouse after IFN-γ treatment.

Gene	*Ciita*	*H2-Ab1*	*H2-Dma*	*Cd74*	*Icam1*
**RQ**	260.5 ± 9.22	**9.19 ± 0.64**	**7.68 ± 0.21**	**65.64 ± 4.12**	**4.37 ± 0.19**
***P* value**	0.0571	0.0022*	0.0022*	0.0022*	0.0022*

IFN-γ treatment was at 200 U/ml. N = 3–8. RQ, relative quantity, is shown as mean ± SE.

*, *P* ≤ 0.05. Bold, significantly changed.

IL-1β and TNF-α have also been shown to cause impaired lacrimal gland secretion [36] and were also significantly elevated in LG from NOD mice (Table 1). However, pilot studies on the effects of IL-1β and TNF-α on gene expression of CTSS and effectors of MHC II-mediated antigen presentation (as in Tables 2 and 4) in cultured mouse LGAC did not show changes comparable to IFN-γ. Moreover, co-treatment of IL-1β or TNF-α with IFN-γ and analysis of the expression of these genes in cultured mouse LGAC did not reveal any synergistic effects. While other cytokines may add to the changes exerted in acinar cell function by IFN-γ, these two which are elevated concomitantly in LG early in disease appeared not to be involved in the processes that are our focus here under our experimental conditions.

### IFN-γ exposure slightly enhances antigen uptake capacity of BALB/c mouse LGAC

**Fig 4:**
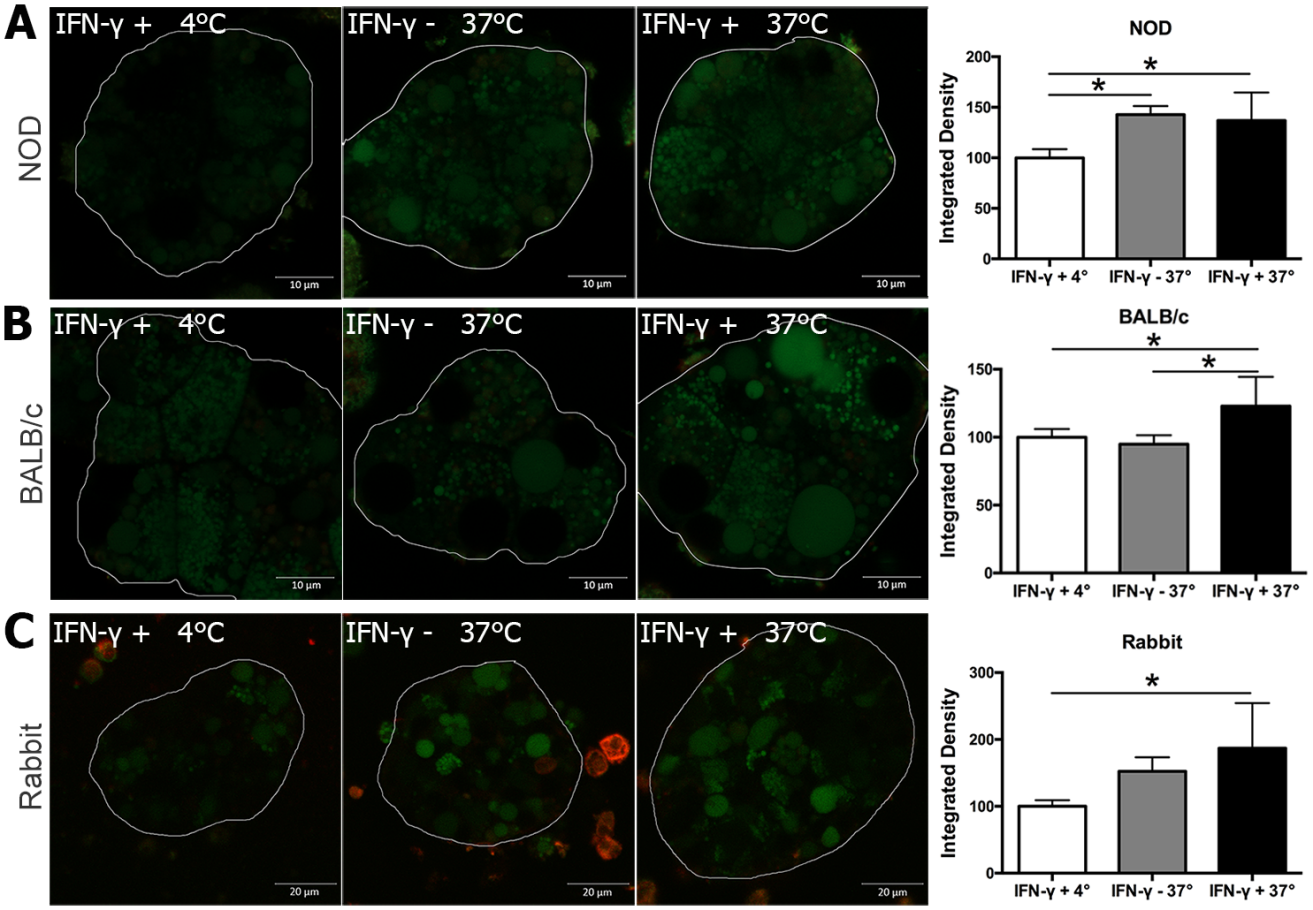
DQ-OVA uptake by mouse and rabbit LGAC. **LGAC from (A)** NOD or (**B**) BALB/c mouse LGAC were cultured with or without 200 U/ml recombinant mouse IFN-γ for 48 hr. (**C**) Rabbit LGAC were cultured with or without 200 ng/ml recombinant rabbit IFN-γ for 48 hr. Medium was changed with 100 μg/ml DQ-OVA and cells were incubated for 60 min at 37°C or 4°C. Cells were washed twice with fresh medium and observed using confocal fluorescence microscopy using appropriate settings for green and red fluorescence as in Methods. **(A)** NOD mouse LGAC showed modestly higher antigen uptake levels without and with IFN-γ. **(B)** BALB/c mouse LGAC showed modestly higher antigen uptake levels with IFN-γ treatment. (**C**) Rabbit LGAC showed modestly higher temperature dependent antigen uptake level. Representative images are shown from three experiments (independent cell preparations). Quantification was done with 4 ROIs from each experiment for each condition.

The uptake and processing of soluble antigen into acidic endosomal compartments is required for antigen presentation. To determine whether the increased gene expression of some components of MHC class II-mediated antigen presentation associated with IFN-γ-treated cultured LGAC was associated with changes in functional antigen processing, we used BODIPY-conjugated DQ-Ovalbumin (DQ-OVA) to assess antigen uptake and processing ability. DQ-OVA is a self-quenched conjugate that exhibits green fluorescence upon proteolytic cleavage. When digested fragments of DQ-OVA accumulate in organelles at high concentration, while the fluorophore may also form excimers that can be visualized using a red light-sensitive long-pass filter [10, 29]. LGAC isolated from male NOD mice aged 12 weeks, when autoimmune dacryoadenitis is established, showed visible antigen uptake capacity independently of IFN-γ, indicating the conversion of these acinar cells to antigen presenting cells in vivo in the disease model prior to any experimental treatments (Fig 4A). A lesser but still significantly higher DQ-OVA uptake capability was seen in BALB/c mouse LGAC treated with but not without IFN-γ (Fig 4B). Rabbit LGAC cultured similarly and treated with IFN-γ also showed an similar modest increase in temperature dependent uptake of DQ-OVA (Fig 4C) although the role of IFN- γ in this increase is less clear since it was not significant relative to untreated LGAC cultured at 37°C.

### Increased IFN-γ levels in tears is correlated with increased expression of CTSS and components of MHC class II–mediated antigen presentation in corneas of male NOD mice

**Fig 5:**
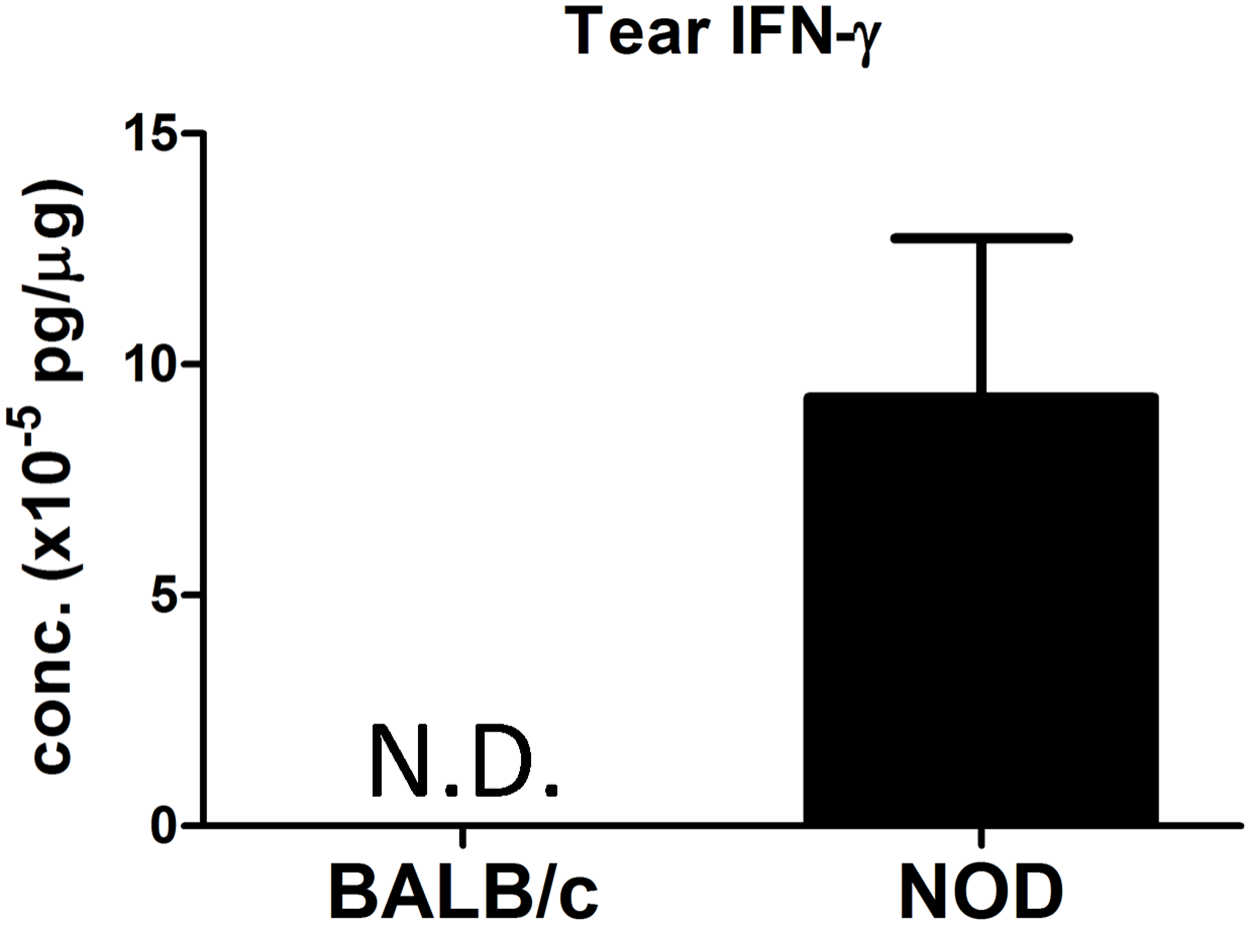
IFN-γ levels are increased in tears from 12-week male NOD mice relative to age-matched male BALB/c mice as determined by MSD^®^ multiplex assay. The IFN-γ concentration is expressed as pg per μg total tear protein. IFN-γ was not detected in BALB/c mice tears (N.D., limit of detection approximately 0.449 × 10^−5^ pg/μg) but was detectable in NOD mice tears. Tears from 4 NOD and 4 BALB/c mice were pooled for each n value to obtain sufficient sample for analysis. 16 BALB/c and 16 NOD male mice, aged 12 weeks, were used for N = 4.

IFN-γ is increased in tears and saliva of SS patients [37–39]. By applying the same multiplex assay, we utilized in NOD mouse LG in Table 1 to tears, we found that IFN-γ levels were significantly higher in tears from male NOD mice compared to levels found in tears of the control strain, male BALB/c mice, which was below the limits of detection (Fig 5). To begin to investigate the relationship of tear IFN-γ to the ocular surface, we isolated corneas from male NOD and BALB/c mice and analyzed gene expression levels of components of MHC class II-mediated antigen presentation (Table 5). The gene expression levels of CTSS, MHC class II, H2-DM molecule, Ii and ICAM1 were all significantly increased in NOD mouse corneas, relative to levels present in BALB/c mouse corneas.

**Table 5:** Changes in gene expression of MHC class II-related molecules in corneas of male NOD mouse compared with BALB/c.

Gene	*Ctss*	*H2-Ab1*	*H2-DMa*	*Cd74*	*Icam1*
**RQ**	**2.78 ± 0.56**	**3.89 ± 1.02**	**2.60 ± 0.16**	**53.03 ± 13.35**	**2.51 ± 0.24**
***P* value**	0.0365*	0.0468*	0.0006*	0.0176*	0.0038*

N = 3. RQ, relative quantity, is shown as mean ± SE.

*, *P* ≤ 0.05. Bold, significantly changed.

### IFN-γ exposure increases expression of CTSS and MHC class II-related molecules and antigen presentation in corneal epithelial cells

**Fig 6:**
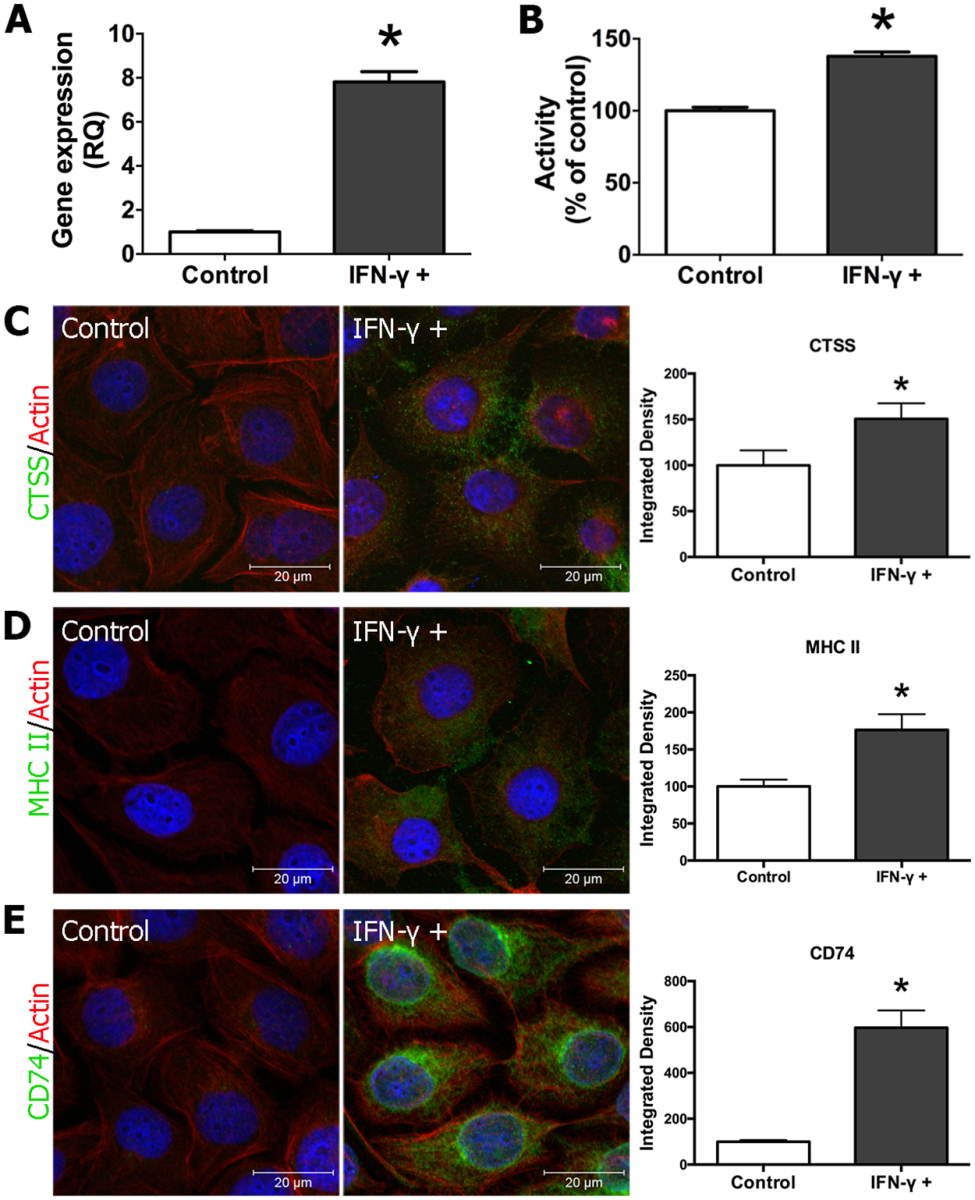
CTSS gene expression, activity and protein expression in HCE-T cells treated with IFN-γ (1 μg/ml) for 48 hr. **(A)** Gene expression of CTSS was increased by 7.8-fold (*P* = 0.0035) in HCE-T treated with IFN-γ. **(B)** The activity of CTSS was increased by 1.37-fold (*P* = 0.0005) in HCE-T cells treated with IFN-γ. Increased CTSS **(C)**, MHC class II (MHC II) **(D)** and invariant chain (CD74) **(E)** protein expression was also detected by indirect immunofluorescence in HCE-T treated with IFN-γ as described above. Representative images are shown from three experiments. Quantification was done as in Methods with 5–10 ROIs from each experiment for each marker.

To determine whether IFN-γ contributes to the observed increased expression of CTSS and components of MHC class II-mediated antigen presentation in corneal epithelial cells, we treated the human cornea epithelial cells transformed with Simian virus-Adeno vector (HCE-T) with IFN-γ in vitro. After 48 hr of exposure to 1 μg/ml of IFN-γ, CTSS gene expression was increased significantly (Fig 6A). CTSS activity in HCE-T lysates was also commensurately increased (Fig 6B) while the increase in CTSS protein expression was detected by immunofluorescence (Fig 6C). MHC class II and invariant chain (CD74) expression were also increased with IFN-γ stimulation (Fig 6D and 6E) as reported previously [40].

**Fig 7:**
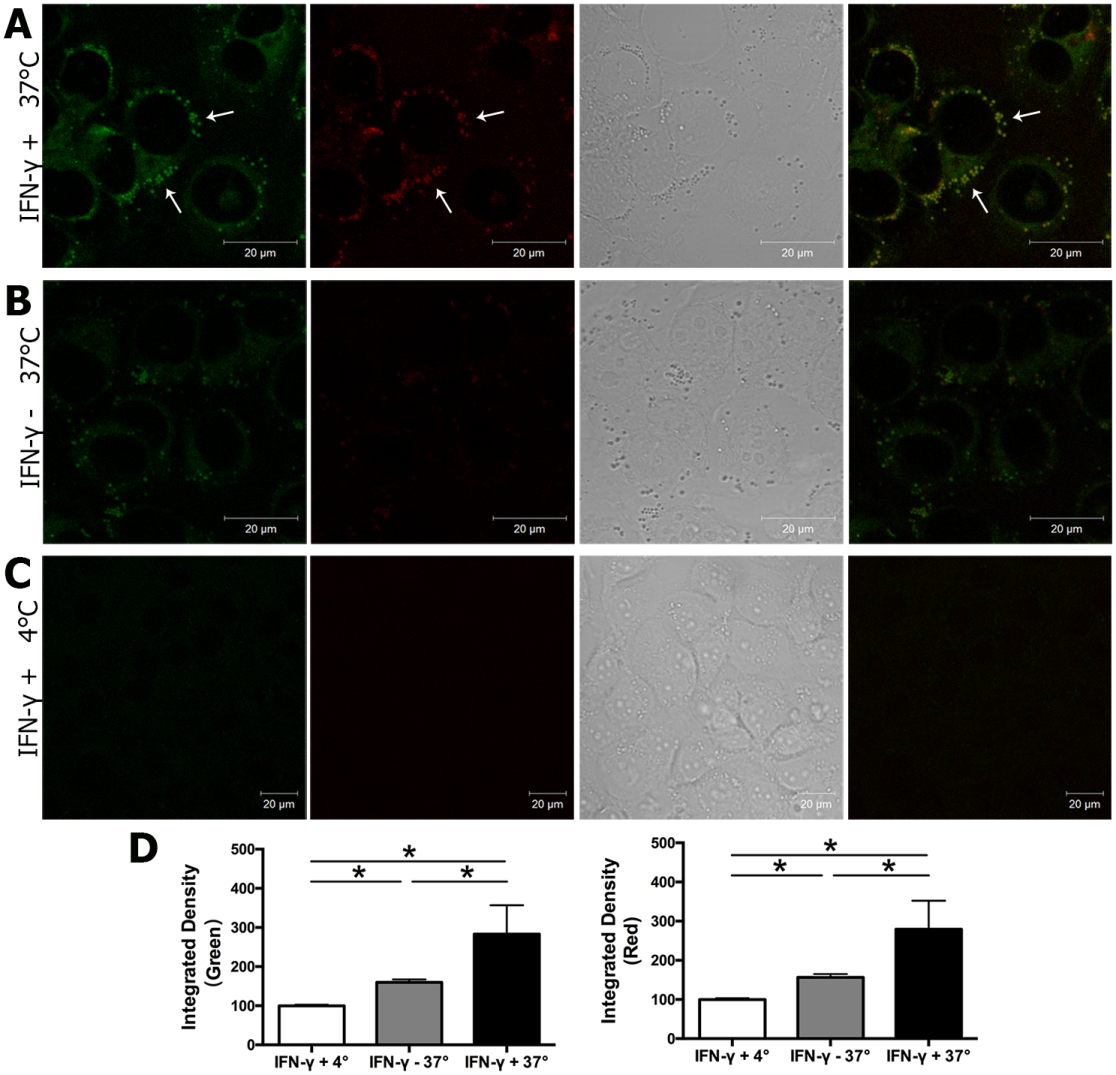
DQ-OVA uptake by HCE-T cells. HCE-T cells were cultured with or without IFN-γ (1 μg/ml) for 48 hr. Medium was changed with 100 μg/ml DQ-OVA and cells were incubated for 60 min at 37°C or 4°C. Cells were washed twice with fresh medium and observed using confocal fluorescence microscopy using appropriate settings for green and red fluorescence as in Methods. Cells treated with IFN-γ **(A)** showed higher green and red fluorescence (arrows) compared with the non IFN-γ treated group **(B)**. Minimal uptake of DQ-OVA was detected at 4°C **(C)**. Representative images are shown from three experiments. Quantification was done as in Methods with at least 16–25 ROIs from each of the 3 experiments for each condition.

To determine whether the increased expression of components of MHC class II-mediated antigen presentation associated with IFN-γ-treated HCE-T cells resulted in enhanced antigen processing ability, we again used DQ-OVA to assess antigen uptake and processing ability. HCE-T cells exposed to IFN-γ-showed enhanced antigen uptake and processing capability as well as the ability to concentrate digested fragments after IFN-γ treatment (Fig 7A and 7B), both hallmarks of antigen activation. No released fluorescence fragment or excimers were observed in the 4°C control group (Fig 7C).

## Discussion

In the current study, we hypothesized that IFN-γ had the potential to elicit some or all of several predicted early changes in LGAC function associated with the early development of SS (Table 6). We verified at time of early development of autoimmune dacryoadenitis in male NOD mice, that IFN-γ was increased markedly both in the LG and in tears. Our first two areas of exploration related to expression and activity of components of the regulated secretory pathway, specifically Rab3D and CTSS. In vitro studies in LGAC showed that IFN-γ exposure reduced Rab3D gene expression and the intensity of Rab3D immunofluorescence detected around apical/luminal membranes. Rab3D participates in exocytosis in secretory epithelial cells, including release of tear proteins from mature secretory vesicles in LGAC. Our previous work showed that loss of Rab3D in LGAC resulted in increased CTSS secretion, perhaps due to compensatory activity of another Rab, Rab27, that directly facilitates CTSS release from secretory vesicles [17]. Reduced Rab3D expression and altered distribution in LG and SG of SS patients [16, 23] is associated with loss of cell polarity and altered tear protein spectra.

**Table 6:** Summary of four putative changes in response to abnormalities in IFN-γ levels in the male NOD LG acinar cells, primary LG acinar cells in vitro and HCE-T cells.

	Male NOD	Primary LGAC	HCE-T
**Altered Rab3D expression and distribution**	+++	++	NA
**Increased stimulated CTSS secretion**	+++	++	ND
**Increased expression of MHC class II mediated antigen presentation machinery**	+++	+	++
**Functional antigen processing**	+	+	++

+++, significant change;

++, moderate change;

+, marginal change;

ND, not determined; NA, not applicable.

IFN-γ also increased the stimulated secretion of CTSS from LGAC, a phenomenon previously linked to decreased Rab3D expression [17]. This is also an established characteristic of SS, confirmed in both NOD mouse [17, 19] and SS patients [18]. The increase in CTSS activity in tears serves as potential biomarker for early diagnosis of SS since it is specifically increased in tears from SS patients compared with patients with other autoimmune or dry eye diseases. As a protease involved in multiple physiological pathways, tear CTSS may compromise ocular surface integrity through diverse mechanisms including matrix degradation and induction of multiple inflammatory pathways [21, 22], thus promoting the ocular surface inflammation that accompanies autoimmune LG inflammation in SS patients. The magnitude of the changes elicited by IFN-γ in vitro on these disease-associated changes in LGAC secretory function, loss of Rab3D and increased expression and secretion of CTSS, did not occur at the magnitude seen in the male NOD mouse LG in vivo at disease onset, suggesting that additional activators or sustained exposure over conditions greater than those achievable in vitro may be required for full induction of these early pathological changes.

The second series of changes in epithelial function that we hypothesized from studies in SG acinar cells, and suggested by initial work in LG, involves increased expression and function of components of MHC class II-mediated antigen presentation including, in an additional context, CTSS as well as ICAM1. Increased expression of MHC class II components and ICAM1 may reflect the first transition whereby these cells begin to participate in the autoimmune inflammatory process. Although constitutive antigen presentation activity is restricted to bone marrow-derived antigen presenting cells including dendritic cells, B cells and macrophages, its expression can be upregulated by pro-inflammatory cytokines in many cells including epithelial cells. The modest IFN-γ-induced antigen uptake function of BALB/c LGAC and IFN-γ-independent antigen uptake function of LGAC isolated from SS disease NOD mice in this study reinforce this point. A role for CTSS in increasing MHC class II-mediated antigen presentation has also been illustrated in studies using CTSS-/- mice [41–46]. Although gene expression studies seemed to indicate increased expression of these effectors in BALB/c mouse LGAC treated with IFN-γ, these changes were not associated with any significant IFN-γ–induced accumulation of protein components of MHC class II-mediated antigen presentation. Moreover, although we have shown that the gene expression of CTSS is elevated by IFN-γ exposure in cultured LGAC (Table 2, S1 Fig) while its stimulated secretion is likewise increased (Fig 1C), its activity in lysates is not measurably increased in parallel (Fig 1A and 1B). This observation might be due to the presence of natural CTSS inhibitory factors in the cells. We tested this hypothesis by incubating human or mouse recombinant CTSS with mouse LGAC lysate. The activity of recombinant mouse CTSS was markedly reduced by exposure to mouse LGAC lysate (S2 Fig).

Cystatin C is the principal endogenous CTSS inhibitor. Its expression is normally downregulated in NOD mouse LG in parallel with increased CTSS activity. However, when we analyzed the cystatin C gene expression levels in mouse LGAC cultured with IFN-γ, there was no change in its expression. In IFN-γ-stimulated macrophages, CTSL activity is downregulated but the intracellular mature protein level is maintained [30], suggesting an upregulation of inhibitor. In immature dendritic cells, insufficient Ii degradation due to the low CTSS activity inhibited by cystatins results in MHC class II accumulation in lysosomes. Maturation of dendritic cells is accompanied by a reduction in cystatin levels and Ii degradation by CTSS [46]. These data all support the existence of additional inhibitory mechanisms for endogenous CTSS activity that are not reversed by 48 hr of IFN-γ-treatment in LGAC, perhaps explaining the failure of LGAC exposed solely to IFN-γ for 48 hr to robustly process and present antigen in vitro.

In addition to the possibility that inhibitory factors of CTSS impair its antigen processing and Ii degradation capabilities, another explanation for the lack of a robust functional antigen processing capability triggered by IFN-γ might be the expression of different signaling pathways in LGAC and SG acinar cells. One study showed that no signs of lymphocytic infiltration were observed in SG of IFN-γ- or IFN-γ receptor-knockout NOD mice at 20 weeks, while lymphocytic infiltration was not reduced in the LG of these knockout mice compared with the parent NOD strain [47]. Consistent with this hypothesis, gene expression of components of MHC class II-mediated antigen processing is increased by IFN-γ, yet there is no detectable commensurate increase in protein expression, suggesting that additional factors that may participate in protein translation, protein half-life or trafficking may be required.

In contrast to studies in cultured LGAC, IFN-γ was highly effective at increasing components of MHC class II mediated antigen presentation and in inducing functional antigen uptake and processing in human corneal epithelial cells. Higher IFN-γ levels are present in tears of patients with dry eye diseases [48]. Elevated tear IFN-γ concentration is correlated with tear hyperosmolarity and development of clinical parameters, including increased ocular surface staining and low Schirmer’s test scores, in evaporative dry eye [49]. We have shown in this study that IFN-γ is also elevated in the tears of a mouse model of SS. The origins of the tear IFN-γ as well as elucidation of additional features of its impact on ocular surface inflammation beyond the changes in corneal epithelial cells observed here are worthy of further investigations.

In summary, we have shown that IFN-γ is highly elevated early in development of autoimmune dacryoadenitis in the LG of a mouse model of SS. Identifying four early potential pathological changes in the secretory acinar cells of the LG as targets of pro-inflammatory cytokines, we show here that IFN-γ was able to alter expression of Rab3D, and to increase CTSS secretion through the regulated secretory pathway (Table 6). IFN-γ induced only a modest increase in functional antigen presentation in LGAC, but a much more robust increase in corneal epithelial cells of the ocular surface (Table 6). These studies collectively suggest a role for IFN-γ in early disease pathology in the development of ocular symptoms in LG and ocular surface in SS.

## Materials and methods

### Mice and Rabbits

NOD mouse breeding pairs were purchased from Taconic (Oxnard, CA) and colonies used in this study were bred in-house. BALB/c mice were obtained from Jackson Laboratories (Sacramento, CA). Female New Zealand White rabbits (1.8–2.2 kg) were obtained from Irish Farms (Norco, CA). All animal procedures were in accordance with the Guiding Principles for the Use of Animals in Research and approved by University of Southern California Institutional Animal Care and Use Committee. Mice were euthanized by intraperitoneal injection with ketamine/xylazine (60–70 mg + 5–10 mg/kg), followed by cervical dislocation. Rabbits were euthanized with ketamine/xylazine (50 mg + 5 mg/kg) followed by lethal injection of sodium pentobarbital (120 mg/kg) prior to removal of LG.

### Materials

Pro-inflammatory Panel 1 (mouse) Kits were from Meso Scale Discovery (MSD^®^) (Rockville, MD). RNA extraction kits were obtained from Qiagen (Hilden, Germany). The reverse transcription kit, primers and master mix for real-time PCR were purchased from Applied Biosystems (Foster City, CA). O.C.T. Compound was obtained from VWR (Radnor, PA). Rabbit anti-Rab3D polyclonal antibody was generated by Antibodies Inc. (Davis, CA) as previously reported [50]. FITC anti-mouse I-Ad polyclonal antibody (#115006) which recognizes g7 and d haplotypes of mouse MHC class II was from Biolegend (San Diego, CA). Goat anti-ICAM-1 polyclonal antibody (#AF796) was from R&D Systems (Minneapolis, MN). Rabbit anti-CIITA polyclonal antibody (#5979) was from Prosci (Fort Collins, CO). Goat anti-HLA-DMa (#14536) and goat anti-CD74 (#20082) polyclonal antibodies were from Santa Cruz Biotechnology (Dallas, TX). Rhodamine phalloidin, Alexa Fluor^®^ 488 and 568 secondary antibodies and the ProLong^®^ Gold Antifade Mounting Medium were from Invitrogen (Carlsbad, CA). Recombinant mouse and human IFN-γ were from EMD Millipore (Darmstadt, Germany). Recombinant mouse IL-1β and TNF-α proteins were purchased from Abcam (Cambridge, United Kingdom). Recombinant mouse CTSS protein was from Novoprotein (Summit, NJ) and recombinant human CTSS was from Biovision Inc. (Milpitas, CA). The mouse CTSS ELISA kit was from LifeSpan BioSciences, Inc. (Seattle, WA). The CTSS Activity Fluorometric Kit was from Biovision Inc. Keratinocyte serum-free medium (KSFM) supplied with human recombinant Epidermal Growth Factor and Bovine Pituitary Extract were from Gibco^®^, Life Technologies (Grand Island, NY). BODIPY-conjugated DQ-OVA was from ThermoFisher SCIENTIFIC (Waltham, MA). All other chemicals were reagent grade and obtained from standard suppliers.

### Multiplex assays on LG and tears

LG were collected from NOD and BALB/c mice and lysed with lysis buffer (150 mM NaCl, 20 nM Tris base pH 7.5, 1 mM EDTA, 1 mM EGTA, 1% Triton X-100) with protease inhibitor cocktail as described previously [19]. One LG from each mouse was lysed with 600 μL buffer. Lysate samples were diluted 1:3 for the assay. The manufacturer’s protocol for the MSD MULTI-SPOT Assay System was followed. The plate was read with an MSD Sector Imager 2400A Imaging System. A standard curve for each cytokine was created.

### Laser capture microdissection

LG were retrieved from NOD and BALB/c mice, embedded in O.C.T. compound, and rapidly frozen with liquid nitrogen removed under RNase-free conditions. The frozen blocks were cryosectioned and collected with membrane-coated slides (PEN; Leica Microsystems, Buffalo Grove, IL) and stained with Hematoxylin. Acinar cells were then cut and collected by a Laser Microdissection Systems (LMD7000, Leica, Buffalo Grove, IL) following the manufacturer’s protocol and as shown in S1 Fig.

### Gene expression assays

RNA was extracted from LGAC or corneas isolated from NOD and BALB/c mice or HCE-T cells, using RNeasy^®^ Plus Micro kits or RNeasy^®^ Plus Mini kits, depending on the sample type. Reverse transcription reactions were performed using TaqMan^®^ Reverse Transcription Reagents to obtain cDNA from RNA. Quantitative polymerase chain reaction (qPCR) was conducted using an ABI 7900HT Fast Real-Time PCR System. 1 μl of RT product (diluted with 8 μL of nuclease-free H2O), 1 μl of the primer and 10 μl of Universal Master Mix were used in each reaction in a total volume of 20 μl. Gapdh (Glyceraldehyde 3-phosphate dehydrogenase) was run as the internal control. The reaction conditions and calculation methods were as described previously [51]. The recorded data were analyzed using the ΔΔCt study calculating function of the ABI software SDS 2.1. The relative quantity (RQ) for a specific mRNA was obtained by calculations using the equations ΔCt = Ct (studied mRNA) − Ct (house-keeping gene mRNA), ΔΔCt = ΔCt (experiment group) − ΔCt (control group), and RQ (experiment group/control group) = 2^-ΔΔCt^.

### Preparation of rabbit and mouse primary LGAC and cytokine treatments

Isolation of rabbit LGAC was as previously described [51]. LG isolated from rabbit were rinsed with Ham’s medium and cut into 1 mm^3^ pieces. The pieces were transferred to an Erlenmeyer flask and incubated sequentially in Ca^2+^- and Mg^2+^-free HBSS supplemented with EDTA and Ham’s medium supplemented with collagenase, hyaluronidase, and DNAse at 37°C. Supernatant was aspirated after each incubation. The cells were pelleted by centrifugation and filtered through a 70 μm cell strainer. The filtrate was carefully layered on top of 5% Ficoll and centrifuged to remove other cells. The pelleted acinar cells were washed with Ham’s and resuspended with Peter’s serum-free culture medium (PCM) [51]. Isolation of mouse LGAC was similar with some modifications. Specifically, 100 μm cell strainers were used instead of the 70 μm strainers used for rabbit. LG from male BALB/c mice (3–4 months old) were collected. Cells were seeded at 2 x 10^6^ cells per well in 12-well plates. For fixation and analysis by indirect immunofluorescence, cells were seeded on coverslips in 12-well plates. For antigen uptake assay, cells were seeded at 6 x 10^6^ in 35 mm-Petri dishes. Plates and Petri dishes were coated with Matrigel^®^. Mouse LGAC were allowed to recover for 2 hr in PCM medium before being treated with pro-inflammatory cytokines. IFN-γ was applied with the indicated dose and durations. Rabbit LGAC were treated with IFN-γ 1 day after the preparation when the formation of acini was initiated.

### HCE-T cell culture and IFN-γ treatment

The HCE-T cell line, transformed with Simian virus 40-Adeno vector [52], was obtained from RIKEN BRC Cell Bank (Japan) (Cat. #RCB2280) and was cultured with KSFM supplemented with human recombinant Epidermal Growth Factor and Bovine Pituitary Extract. Cells between passages 4–9 were used for experiments. Cell starvation was performed before IFN-γ treatment by culturing cells in KSFM only for 16–18 hr after which active human recombinant IFN-γ (1 μg/ml) was added to the full culture medium for 48 hr.

### Determination of CTSS activity in cell lysates

CTSS activity in LGAC and HCE-T lysates was determined with the assay kits described above, according to the manufacturer’s instructions, and the enzymatic reaction was incubated at 37°C for 2 hr. The quantity of the resulting fluorescent products was measured by BioTek Synergy H1 Hybrid Multi-Mode Microplate Reader. Activity of CTSS was measured as relative fluorescence units per microgram protein. Total tear protein concentration was measured using the BCA protein assay. For the assessment of stability of CTSS in mouse LGAC lysate in S2 Fig, 0.5 μg human or mouse recombinant CTSS was used.

### Immunofluorescence labeling of cells and LG tissue and imaging and quantitative analysis by confocal fluorescence microscopy

LG from 12-week male NOD mice were retrieved and fixed in 4% paraformaldehyde for 2–3 hr and then immersed in 30% sucrose at 4°C overnight. Fixed LG were embedded in O.C.T. compound and flash-frozen in liquid nitrogen. The blocks were cryosectioned at 5 μm thickness and mounted on glass slides. The sections were permeabilized with 0.1% Triton X-100 for 10 min. Primary BALB/c LGAC and HCE-T cells were fixed and permeabilized with a -20°C acetone and methanol mixture (1:1) for 20 min. After blocking with 1% BSA for 1 hr, tissue sections and cells were incubated with primary and secondary antibodies. After each incubation, samples were rinsed 3 times with PBS. Finally, samples were mounted with ProLong Gold Antifade mounting medium and imaged by confocal fluorescence microscopy using a Zeiss LSM 510 Meta NLO equipped with Argon, red HeNe, and green HeNe lasers and a Coherent Chameleon Ti-Sapphire laser (Carl Zeiss, Thornwood, NY). Quantification of the signals was done using ImageJ. Regions of interest (ROI) were selected from each image and multiple images were taken for each sample. Integrated density was obtained and used for statistics.

### Antigen uptake and processing

HCE-T cells and rabbit LGAC treated with or without IFN-γ for 48 hr were incubated with 100 μg/ml DQ-OVA for 60 min at 37°C or 4°C (control) and then washed twice with medium. The uptake and digest of the tracer ovalbumin was imaged by confocal fluorescence microscopy using Argon 488 excitation and BP 500–550 emission filter for green fluorescence and HeNe1 543 excitation and BP 565–615 emission filter for red fluorescence. Quantification of the signals was done using ImageJ. Regions of interest (ROI) were selected from each image and multiple images were taken for each sample. Integrated density was obtained and used for statistics.

### Statistics

Data were assumed to have a non-normal distribution unless it passed the Kolmogorov-Smirnov, D’Agostino and Person omnibus, and Shapiro-Wilk normality tests. Data analysis was conducted to compare between sets using either a Student’s unpaired two-tailed t-test (for comparison between NOD and BALB/c strains), a paired two-tailed t-test (for comparison between treated and non-treated cells) for normally distributed samples, or Mann-Whitney test for non-normally distributed samples, as appropriate. The criterion for significance was *P* ≤ 0.05.

## Supporting information

S1 Table Changes in gene expression of CTSS, MHC class II-related molecules in rabbit primary LG acinar cells after IFN-γ treatment. (DOCX)

S1 Fig Example image of acinar cell isolation by laser capture microdissection. A NOD mouse LG section is shown before and after laser capture microdissection.
(TIF)

S2 Fig Mouse recombinant CTSS was degraded by BALB/c LGAC lysate. Representative CTSS activity in the presence of endogenous proteins in LGAC lysate from BALB/c mouse LG acinar cells. The actual value is the amount of CTSS activity observed when a given amount of active human (huCTSS) or mouse (msCTSS) recombinant CTSS was added to the cell lysate. The theoretical value is the sum of the CTSS values in lysates and the active huCTSS or msCTSS when measured individually. Data are presented as relative values to the theoretical value. N = 3. *, *P* = 0.0009.
(TIFF)

## Abbreviations

CCh: carbachol
CIITA: MHC class II transactivator
CTSS: cathepsin S
DQ-OVA: BODIPY-conjugated DQ-Ovalbumin
HCE-T: human cornea epithelial cells transformed with Simian virus-Adeno vector
HLA-DM/H2-DM: human/mouse leukocyte antigen DM
ICAM1: Intercellular adhesion molecule-1
IFN: interferon
Ii/CD74: invariant chain
IL: interleukin
LG: lacrimal glands
LGAC: lacrimal gland acinar cells
MHC: major histocompatibility complex
NOD: non-obese diabetic
SE: standard error of the mean
SG: salivary glands
SS: Sjögren’s Syndrome
TNF: tumor necrosis factor
