# Supplementary material for: Interferon-γ treatment in vitro elicits some of the changes in cathepsin S and antigen presentation characteristic of lacrimal glands and corneas from the NOD mouse model of Sjögren’s Syndrome
Source: PLoS One. 2017 Sep 13;12(9):e0184781. doi: 10.1371/journal.pone.0184781 (PMC5597228; doi:10.1371/journal.pone.0184781)
Supplement: S1 Table — (DOCX) [file pone.0184781.s001.docx]

| **S1 Table. Changes in gene expression of CTSS, MHC class II-related molecules in rabbit primary LG acinar cells after IFN-γ treatment** | | | | |
| --- | --- | --- | --- | --- |
| **Gene** | | ***CTSS*** | ***RLA-DR-ALPHA*** | ***CST3*** |
| **24 hr** | **RQ** | **8.065** | **4.985** | 1.079 |
|  | ***P* value** | **0.0167*** | **0.0121*** | 0.3758 |
| **48 hr** | **RQ** | **9.195** | **1.726** | 0.999 |
|  | ***P* value** | **0.0167*** | **0.0485*** | 0.9999 |
| IFN-γ treatment was at 200 ng/ml. *CST3*, cystatin C. N=3-8. RQ, relative quantity. *****, *P* ≤ 0.05. Bold, significantly changed. We were unable to measure other genes of interest because of the lack of available primers in rabbit. | | | | |
