# Supplementary figures and images for: Interferon-γ treatment in vitro elicits some of the changes in cathepsin S and antigen presentation characteristic of lacrimal glands and corneas from the NOD mouse model of Sjögren’s Syndrome
Source: PLoS One. 2017 Sep 13;12(9):e0184781. doi: 10.1371/journal.pone.0184781 (PMC5597228; doi:10.1371/journal.pone.0184781)

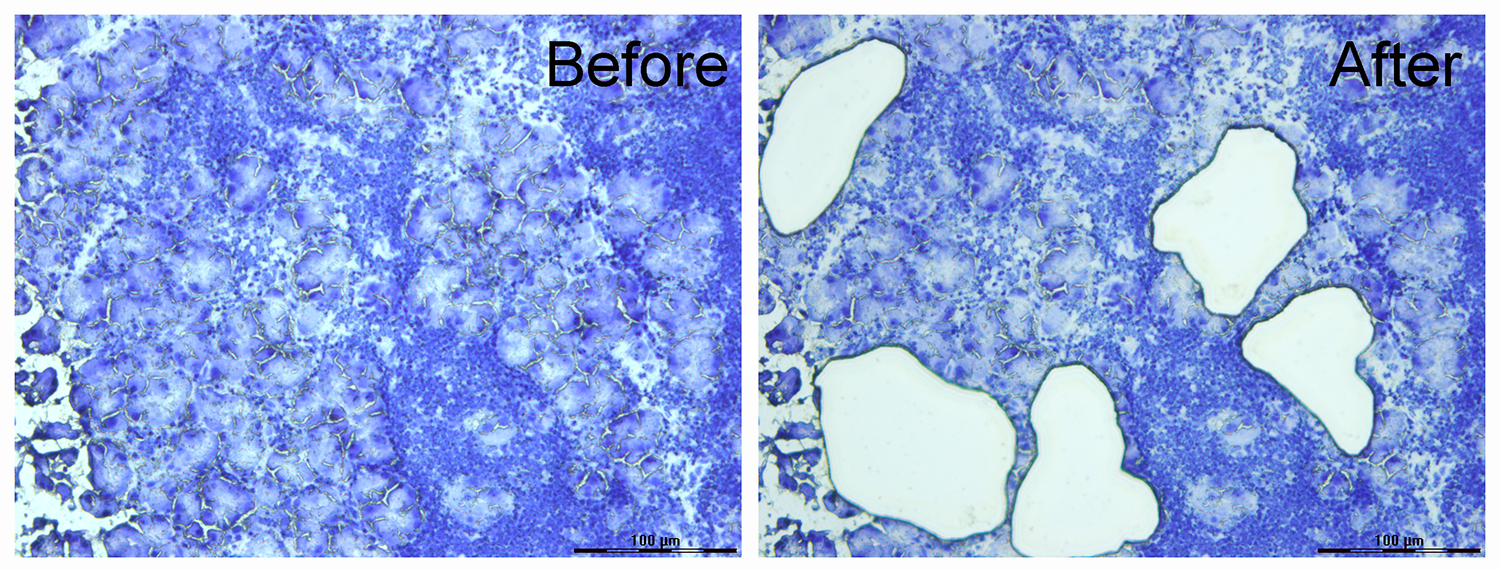

Supplement: S1 Fig — A NOD mouse LG section is shown before and after laser capture microdissection. (TIF) [file pone.0184781.s002.tif]

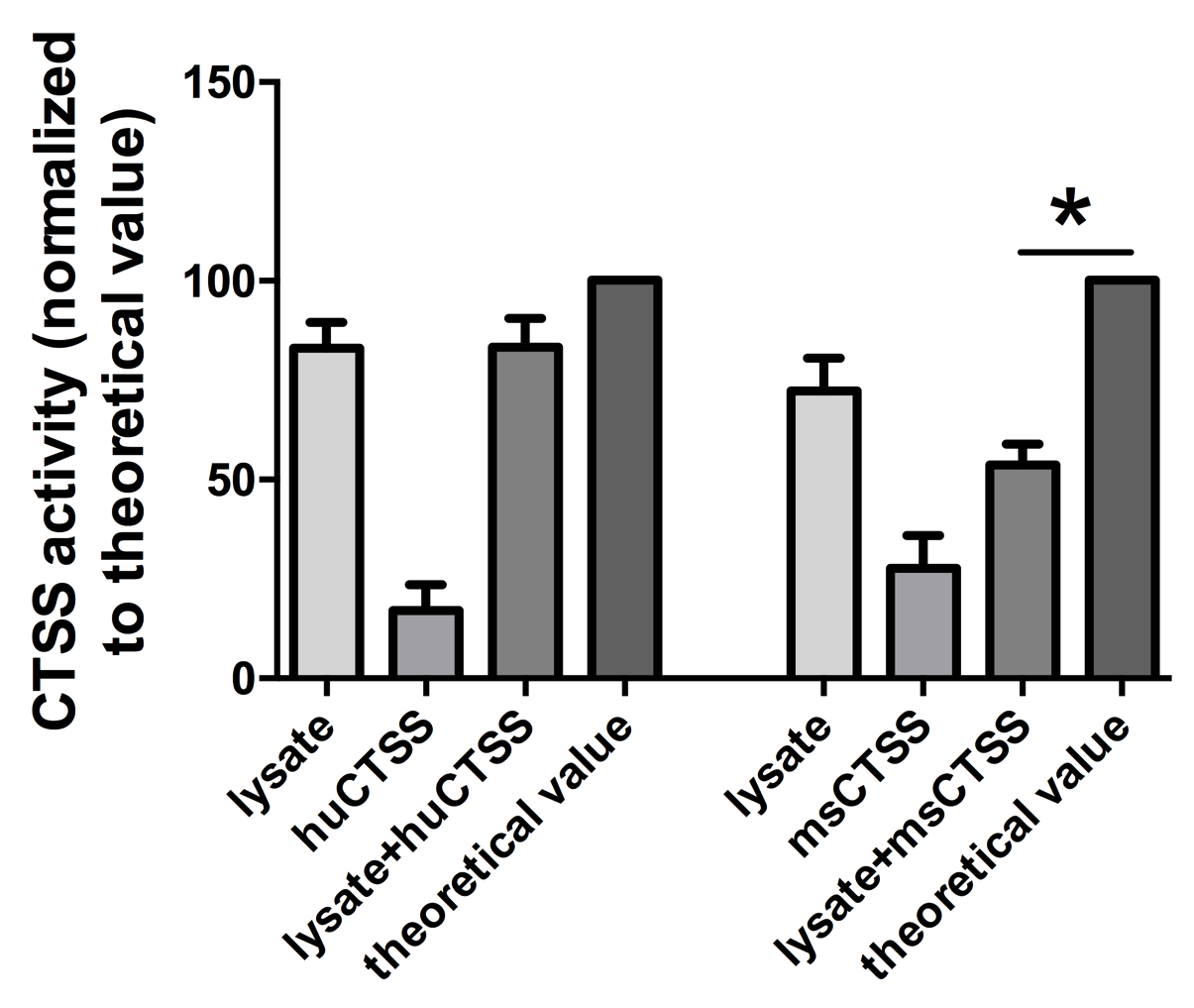

Supplement: S2 Fig — Representative CTSS activity in the presence of endogenous proteins in LGAC lysate from BALB/c mouse LG acinar cells. The actual value is the amount of CTSS activity observed when a given amount of active human (huCTSS) or mouse (msCTSS) recombinant CTSS was added to the cell lysate. The theoretical value is the sum of the CTSS values in lysates and the active huCTSS or msCTSS when measured individually. Data are presented as relative values to the theoretical value. N = 3. *, P = 0.0009. (TIFF) [file pone.0184781.s003.tiff]
